# Space, time and aliens: charting the dynamic structure of Galápagos pollination networks

**DOI:** 10.1093/aobpla/plv068

**Published:** 2015-06-23

**Authors:** Anna Traveset, Susana Chamorro, Jens M. Olesen, Ruben Heleno

**Affiliations:** 1 Laboratorio Internacional de Cambio Global (LINC-Global), Institut Mediterrani d'Estudis Avançats (CSIC-UIB), C/Miquel Marqués 21, 07190-Esporles, Mallorca, Balearic Islands, Spain; 2 Department of Bioscience, Aarhus University, Ny Munkegade 114, DK-8000 Aarhus C, Denmark; 3 Centre for Functional Ecology, Department of Life Sciences, University of Coimbra, Calçada Martim de Freitas, 3000-456 Coimbra, Portugal; 4 Present address: Universidad Internacional SEK, Facultad de Ciencias Ambientales, Calle Alberto Einstein y 5ta transversal, Quito, Ecuador

**Keywords:** Alien plants, alien pollinators, biological invasions, global change, mutualistic interactions, oceanic islands

## Abstract

Oceanic archipelagos are threatened by the introduction of alien species which can severely disrupt the structure, function and stability of native communities. Here we investigated the pollination interactions in the two most disturbed Galápagos Islands, comparing the three main habitats and the two seasons, and assessing the impacts of alien plant invasions on network structure. We found that the pollination network structure was rather consistent between the two islands, but differed across habitats and seasons. Overall, the arid zone had the largest networks and highest species generalization levels whereas either the transition between habitats or the humid habitat showed lower values. Our data suggest that alien plants integrate easily into the communities, but with low impact on overall network structure, except for an increase in network selectiveness. The humid zone showed the highest nestedness and the lowest modularity, which might be explained by the low species diversity and the higher incidence of alien plants in this habitat. Both pollinators and plants were also more generalized in the hot season, when networks showed to be more nested. Alien species (both plants and pollinators) represented a high fraction (∼56 %) of the total number of interactions in the networks. It is thus likely that, in spite of the overall weak effect we found of alien plant invasion on pollination network structure, these introduced species influence the reproductive success of native ones, and by doing so, they affect the functioning of the community. This certainly deserves further investigation.

## Abstract (precis)

Alien species can severely disrupt the structure, function and stability of native communities. We evaluated the structure of pollination networks in the three main habitats and in the two seasons on the two most disturbed Galápagos Islands, and investigated how such structure is influenced by invasive plants. Alien plants integrate easily into the communities, but show low impact on overall network structure, except for an increase in network selectiveness. The highly invaded and low diversity humid zone showed the highest nestedness and the lowest modularity. Both pollinators and plants were more generalized during the hot season, when most plants were flowering and networks became more nested.

## Introduction

Sexual reproduction is an essential step for the life cycle of most plant species, and is chiefly limited by the quantity and quality of pollen grains arriving to their stigmas (Ashman *et al*. 2004). Pollination is thus a critical step in plant reproduction, and many animals, mostly insects, have a vital role in facilitating this step in ∼90 % of the worlds' plant species (Ollerton *et al*. 2011).

Islands harbour a disproportionate part of the worlds' biological diversity and are particularly rich in endemic and threatened species (Sax and Gaines 2008). Oceanic islands, in particular, are generally characterized by low insect diversity (Gillespie and Roderick 2002) and simplified pollination networks when compared with mainland systems (Olesen and Jordano 2002; Traveset *et al*. 2015*b*). This low abundance and diversity of pollinators on islands is likely to translate into reduced pollinator redundancy, potentially leading to highly vulnerable communities when faced with disturbances, e.g. El Niño Southern Oscillations (Traveset and Richardson 2006). Ecological networks offer a most valuable solution to evaluate the overall changes in community structure and function as a response to disturbances affecting species composition (Bascompte 2010; Heleno *et al*. 2014). For example, in order to survive in such low diversity ecosystems, some animal species that successfully colonize isolated islands tend to broaden their trophic niches, thus interacting with more species (mutualistic partners or prey) than their continental counterparts (Carlquist 1974; Olesen *et al*. 2002). This expansion of the feeding niche can characterize entire island communities, a phenomena coined ‘interaction release’ and that tends to have a stabilizing effect on insular interaction networks (Traveset *et al*. 2015*a*). Apart from their low diversity and high generalization when compared with continental communities, oceanic island interaction networks tend to be characterized by an increased nestedness, i.e. an ordered interaction distribution pattern where specialist species interact with specific sub-sets of the partners of most generalist species (Olesen and Jordano 2002; Padrón *et al*. 2009; Kaiser-Bunbury *et al*. 2010; Traveset *et al*. 2013). While increased generalization and nestedness may increase network stability (Sebastián-González *et al*. 2015), overall low biodiversity and the existence of small endemic populations suggest high species vulnerability at least to some specific sources of disturbance, such as invasive species (Berglund *et al*. 2009; Traveset and Richardson 2014).

In this study we focus on the impacts of alien plants on pollination networks. Biological invasions are a growing threat to the worlds' biodiversity (Lambertini *et al*. 2011) and particularly worrying on oceanic islands, where the arrival of alien species frequently triggers serious disruptive effects on the intricate network of interactions established between native species throughout their shared evolutionary history (Kaiser-Bunbury *et al*. 2011; Traveset *et al*. 2014). Specifically, applying a network approach to frame biological invasions at the community level is particularly suitable for clarifying how invasive species can integrate into the existing interaction networks, the likely consequences for community structure, and the consequences for the most vulnerable species (Memmott *et al*. 2007; Bascompte 2009; Traveset and Richardson 2014). In fact, if pollination networks vary naturally in space and time, it is likely that opportunities for alien pollinators and plants to ‘infiltrate’ those networks will also vary in space and time. Thus, aliens may find particularly favourable biotic and abiotic conditions under which their integration into the native communities (and potential invasion) is more likely. Recent studies have begun to evaluate the temporal and spatial variability of pollination network structure (e.g. Olesen *et al*. 2008; Petanidou *et al*. 2008; Dupont *et al*. 2009); however, we are only starting to understand such patterns, how they are related to each other and, particularly, how spatio-temporal dynamics might affect the capacity of alien species to infiltrate into and impact pollination networks. For example, an invasion might be more likely during a particular season, year (e.g. during particularly wet years), or in certain habitats.

As in most archipelagos throughout the World, the number of alien species in the Galapagos began to accumulate even before the first permanent human settlement of the islands, increased exponentially over the last 50 years in step with increasing human pressure (Tye 2006) and currently forms over 60 % of the vascular flora (Jaramillo *et al*. 2014). Indeed, alien species, both plants and animals, are generally considered the main threat to the conservation of the unique Galápagos biodiversity (Bensted-Smith 2002), and predicting the effects of alien plants and pollinators on the reproduction of native vegetation is a major conservation and scientific goal (Tapia *et al*. 2009; Traveset *et al*. 2013). A recent compilation of plant–animal pollination interactions retrieved data from 38 studies published in the last 100 years in highly scattered literature (Chamorro *et al*. 2012). This study concluded that most interactions were documented by observations highly limited in space and time, and thus identified strong biases in the sampling effort dedicated to different islands, times of day, focal plants and functional groups of visitors, reducing our ability to derive solid generalizations from these incomplete datasets (Chamorro *et al*. 2012).

While alien invasive plants may have a direct negative effect on native plants due to direct competition for space (Magee *et al*. 2001), repercussions may also cascade throughout the entire network of biological interactions of an island or archipelago without necessarily leading to local extinction of native species (Jäger *et al*. 2009). Such a disturbance scenario can be better understood with a network approach (Traveset *et al*. 2013), such as the one we apply here.

Human pressure is not evenly distributed across the islands but is heavily concentrated on the two large central islands: Santa Cruz, the most populous island, and San Cristóbal, which holds the administrative capital of the archipelago. Human developments are restricted to a small proportion of each island's area; however, an extensive use of the transition and highland zones for agriculture boosted the number of alien plant species. Thus, these two islands offer suitable models to improve our understanding of the disruptive effect of alien species on the native interaction networks of the Galápagos and to forecast short- and mid-term impacts on the islands with low human presence (Isabela and Floreana), and long-term impacts on the most pristine uninhabited islands. The main objective of this study was thus to assess the spatio-temporal variation of pollination interactions in the two most disturbed Galápagos islands and to determine whether and how alien plants may modify such interaction patterns. In addition, we investigated if alien species (both animals and plants) differ from endemic and non-endemic natives in their integration into the pollination networks.

## Methods

### Study sites

**Figure 1.**
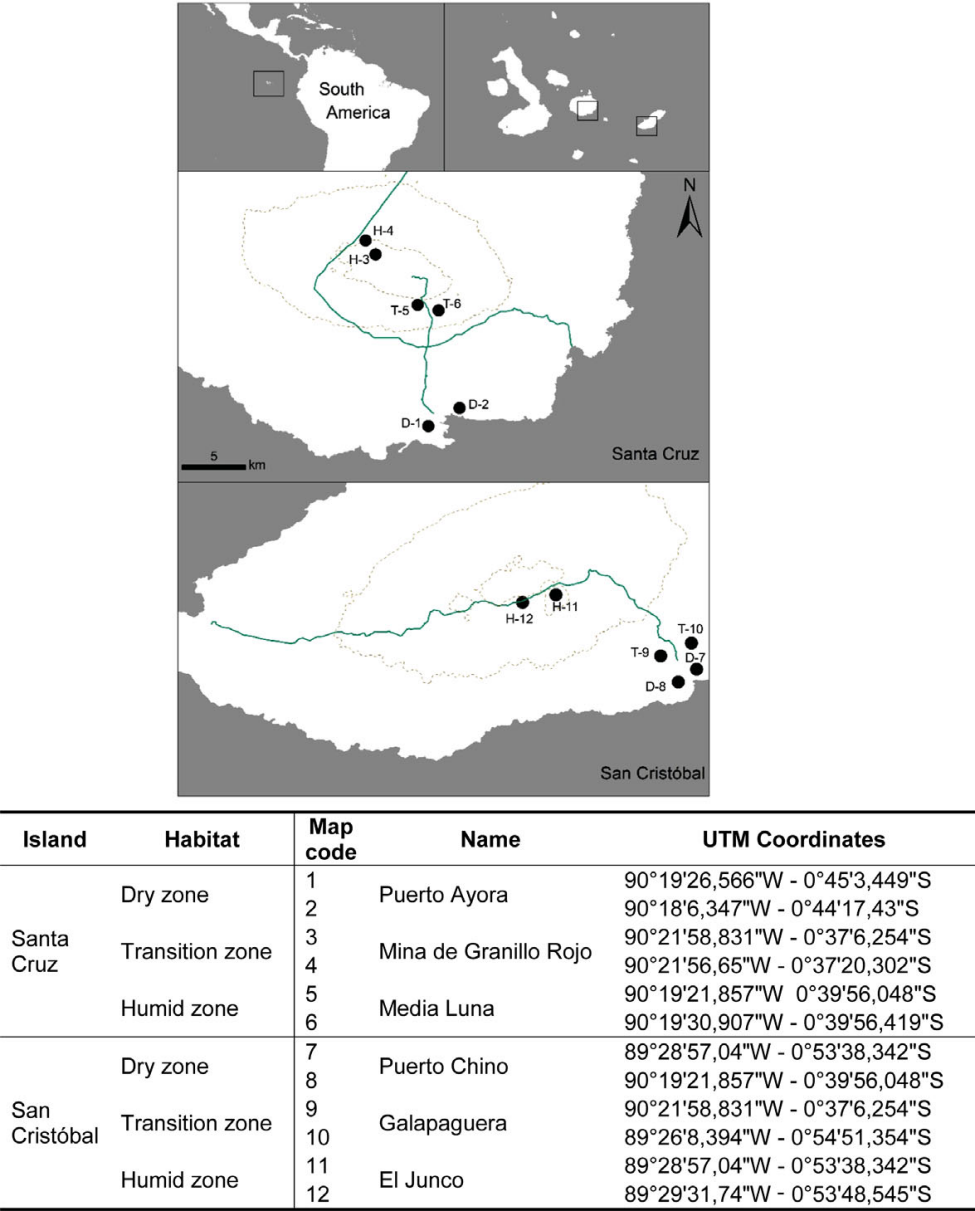
The location of field sites on the islands of Santa Cruz and San Cristóbal in the Galápagos. Contour lines indicate the 300 m and 600 m isoclines. D, Dry lowland sites; T, transition habitat sites; and H, humid highland sites.

The Galápagos archipelago lies at the Equator in the Pacific Ocean, 960 km west of mainland Ecuador (Fig. 1). The archipelago is currently formed by 13 islands larger than 10 km^2^, which were formed by volcanic activity between 0.035 and 4.0 My ago (Poulakakis *et al*. 2012), some of them having been merged in the past due to sea level fluctuations (Ali and Aitchison 2014).

The Galápagos vegetation is marked by strong zonation associated with altitude, with 60 % of the islands' surfaces being markedly dry (Trueman and d'Ozouville 2010). This dry zone occupies the lowlands of all islands and holds most plant diversity and endemic species (McMullen 1999; Guézou *et al*. 2010). The humid zone is restricted to the highlands of the six islands higher than 600 m and is dominated by large patches of the endemic tree *Miconia robinsoniana*, and by woodlands of 16 endemic species of arboreal Asteraceae (*Scalesia* spp.) (McMullen 1999). These two zones are separated by a transition zone characterized by closed mixed forest dominated by several native trees and shrubs including *Zanthoxylum fagara* and *Tournefortia* spp.

The Galápagos climate is characterized by two seasons. The hot/wet season, from January to May, is associated with frequent rain throughout the islands (Trueman and d'Ozouville 2010), and is the time in which most plants flower (Traveset *et al*. 2013) and fruit (Heleno *et al*. 2013*a*). In contrast, the cold/dry season, between June and December, is characterized by virtually no precipitation in the lowlands (Ziegler 1995; Trueman and d'Ozouville 2010). A permanent drizzle generates an evergreen humid habitat in the highest part of the tallest islands, including the two islands included in this study.

This study was conducted at 12 sites on the islands of Santa Cruz and San Cristóbal (Fig. 1). These two islands are highly comparable in terms of area (986 vs. 558 km^2^), elevation (864 m vs. 735 m above sea level), and latitude (0°29′–0.46′S vs. 0°40′–0°56′S, respectively). San Cristóbal is older (max. age = 4.0 My) than Santa Cruz (2.3 My) and is also more isolated from other islands (Fig. 1). Due to the difficulty in finding pristine sites that are accessible from the populated areas in Galápagos, a classical paired experimental design, comparing invaded and uninvaded sites was not possible. Instead, we evaluated the impact of invasion level by quantifying the proportions of alien plants among the 12 selected sites. The invasion level was estimated as the proportion of alien flowers at each site, based on counts of all alien and native flowers at each site, and it ranged from 0 to 73 %. We did not select sites where the native vegetation has been completely replaced by invasive plants such as *Psidium guava*, *Rubus nivaeus*, *Syzygium jambos,* but rather tried to select sites as diverse as possible.

### Pollination observations

Data were collected from 12 sites using a hierarchical design (Fig. 1) including the two most human-populated islands (Santa Cruz and San Cristóbal), and the two most widespread habitats (dry lowland and humid highland) and their transition zone. Sites were sampled during two seasons (wet/hot and dry/cold).

At each site, regular focal flower censuses were performed to quantify the contact of flying animals, mostly insects, with the reproductive organs of open flowers. Each site was visited every other week, when climatic conditions allowed, from March to May (rainy season) and from July to September (dry season) in 2010. In 2011, the 12 sites were re-sampled but only during the flowering peak (hot season); this year, sampling took place between January and May. On each sampling day, flower diversity and abundance were measured along a 500 × 6 m transect (i.e. 3000 m^2^), as follows: (i) all species with open flowers were identified, (ii) the number of individuals of each species was counted, (iii) for each species, the number of flowers on two individuals having an average flower display was counted and (iv) the number of open flowers for each species in the transect was extrapolated from the number of flowers per individual × number of individuals. For species with tightly clustered inflorescences (e.g. the capitula of Asteraceae) we scored each inflorescence as a flower, as this is the ecologically relevant unit visited by pollinators.

Flower-visitors were censused during periods of 10 min in front of target plants (∼1 m away). On each census day, all species with open flowers (regardless of their anthesis stage and nectar production) were observed for at least two non-consecutive periods between 06:00 h (sunrise) and 22:00 h. Nocturnal censuses were made by means of red (low energy) l.e.d. headlights to avoid affecting insects. Species were arbitrarily selected for the different time periods to avoid censusing the same species always at similar times of the day. In each census period we recorded: (i) identity of the flowering plant species, (ii) number of open flowers observed on each individual plant (often only one branch was observed in the case of shrubs or trees), (iii) identity of each flower-visitor, (iv) number of individuals of each species visiting flowers and (v) number of flowers visited by each individual flower visitor. The sampling protocol resulted in 1145 h of flower visitation censuses (on a total of 283 287 flowers) of 119 plant species of which 36 are introduced in the Galápagos. Plant identifications followed McMullen (1999) and information available at the Charles Darwin Foundation Herbarium (Jaramillo *et al*. 2014). Insects that could not be identified in the field were collected for further identification by taxonomists at the Charles Darwin Entomological Collection (see Acknowledgements). Note that here we use the term ‘pollinator’ regardless of its effectiveness in the pollination process, as we do not know whether flower visitation results in pollination.

### Network and statistical analyses

For each site (12 sites), season and year, we built a quantitative plant–pollinator interaction matrix. Thus, we ended up with a total of 36 matrices: 24 for 2010 and 12 (only hot season) for 2011. In each matrix, interactions were quantified by means of visitation frequency, expressed as the total number of visits to the flowers of each species per unit of time, standardized by the number of flowers observed in each census and by the overall flower abundance of each species (Castro-Urgal *et al*. 2012). From each matrix, we obtained 10 parameters commonly used to describe network structure. Seven are network-level parameters: species richness (S); connectance (C); interaction strength asymmetry (IAc), corrected for network asymmetry; interaction evenness (IE); complementary network specialization $(H_{2}^{\prime });$ nestedness [weighted nestedness based on overlap and decreasing fill (WNODF)]; and modularity (M). The other three are species-level parameters: linkage level (*L*), species specialization (*d*′) and species strength (*st*), for both pollinators and plants. Definitions of each of these parameters can be found in **Appendix S1****[see Supporting Information]**. All parameters were computed for the 36 networks using the package ‘bipartite’ v. 2.00 (Dormann *et al*. 2009) in R *v*. 3.1.0 (R Development Core Team 2014), the software NODF *v*. 2.0 (nestedness based on overlap and decreasing fill; Almeida-Neto and Ulrich 2011) (http://www.keib.umk.pl/nodf/) for the calculation of WNODF, and the software MODULAR (Marquitti *et al*. 2014) for the computation of modularity.

We used generalized linear models (GLMs) to test for a significant variation in the network level parameters between islands, habitats, seasons and in relation to invasion level. The fitted models, one for each parameter as a response variable, thus included all four predictors. Species richness (count data) followed a Poisson distribution, and was thus approached by a log link function (Zuur *et al*. 2009), whereas the rest of parameters (all continuous) were approached by the identity link function. Network size (N) was included as a covariate in all models, except for S (directly related to N) and $H_{2}^{\prime }$ (known to be independent of N; Blüthgen *et al*. 2007). For the species-level parameters, we fitted generalized linear mixed models (GLMMs), one model for each parameter as a response variable, and included site (i.e. network) as a random factor to prevent any possible effect of pseudo-replication (as species coexisting in the same site are not independent from each other). The linkage level was fitted to a Poisson distribution and approached by a log link function whereas *d*′ and *st* followed normal distributions and were approached by the identity function. The dredge function in the MuMIn (Multi-model inference) package v. 1.10.5 in R v. 3.1.0 (R Development Core Team 2014) was used to select the best model, i.e. best random and fixed structure of the model for each metric, according to the corrected Akaike's Information Criterion (AIC) (Zuur *et al*. 2009). In order to determine the differences among species, either plants or animals, of different origin (aliens, endemic and non-endemic natives), separate tests were performed with a subset of the data excluding those species whose origin was unknown. For plants, only one unidentified species from the Fabaceae family was excluded from this analysis, whereas for animals (mostly insects), origin was unknown for 83 species (out of the 212 recorded on the flowers; **see Supporting Information—Tables S1 and S2**) and thus the dataset included the remaining 129 species. Origin of insect pollinators was obtained from the Charles Darwin Foundation database (http://www.darwinfoundation.org/datazone/checklists). All analyses were performed using functions *lme* and *lmer* implemented in package *lme4* in the R package v. 3.1.0 (R Development Core Team 2014).

## Results

We recorded a total of 11 125 visits (one individual visitor visiting one flower) by 212 animal species (57 alien to the islands) to the flowers of 111 plant species (32 alien) **[see Supporting Information—Fig. S1 and Tables S1 and S2]**. Except for three species of birds (*Geospiza scandens*, *G. fuliginosa* and *Setophaga petechia*) and one species of lava lizard (*Microlophus bivittatus*), all flower visitors in our networks were insects. The insects belonged to the following taxonomic groups, in order of species richness: Diptera (63 spp.), Lepidoptera (52), Hymenoptera (41), Coleoptera (29), Hemiptera (16), Orthoptera (5), Odonata (1), Collembola (1) and Thysanoptera (1). Overall, 1214 unique, i.e. species-specific, interactions were recorded, of which the majority (43.8 %) corresponded to those between native plants and animals. One-third (33.7 %) of the interactions was found between native plants and alien insects, and alien plants were visited by native and alien insects on 13.5 and 9.0 % of the interactions recorded, respectively.

The proportion of alien plant species was greater on Santa Cruz (27 % overall average) than San Cristóbal (19 %). On Santa Cruz, the number of alien plant species was highest in the humid habitats (40 %), whereas in San Cristóbal most aliens were located in the transition zone (where they represented ∼23 % of the plant species) and in the humid zone (19 %). The frequency of alien plant species was fairly consistent between the two seasons, on both islands and in all three habitats (Table 1). The proportion of alien insect species recorded on the flowers was similar on the two islands, representing an average of 38 % of the total number of insect species. Unlike plants, the frequency of alien insects varied throughout the year and across habitats; on both islands, the highest fraction of alien insect species was found in the cold season in the arid and transition zones (Table 1).

**Table 1.** Frequency of alien plants and pollinators in the 12 study communities (networks).

Island	Habitat/zone	Season	Total plants	% alien plants	Total pollinators	% alien pollinators
Santa Cruz	Arid	Hot	29	3.45	50	42.00
Santa Cruz	Transition	Hot	26	23.08	57	42.11
Santa Cruz	Humid	Hot	26	38.46	46	41.30
Santa Cruz	Arid	Cold	9	11.11	17	58.82
Santa Cruz	Transition	Cold	11	18.18	10	40.00
Santa Cruz	Humid	Cold	15	46.67	10	30.00
San Cristóbal	Arid	Hot	18	5.56	50	44.00
San Cristóbal	Transition	Hot	15	20.00	24	41.67
San Cristóbal	Humid	Hot	14	21.43	29	34.48
San Cristóbal	Arid	Cold	8	0.00	18	55.56
San Cristóbal	Transition	Cold	11	27.27	15	60.00
San Cristóbal	Humid	Cold	6	16.67	10	20.00

### Spatio-temporal patterns at the network level

**Figure 2.**
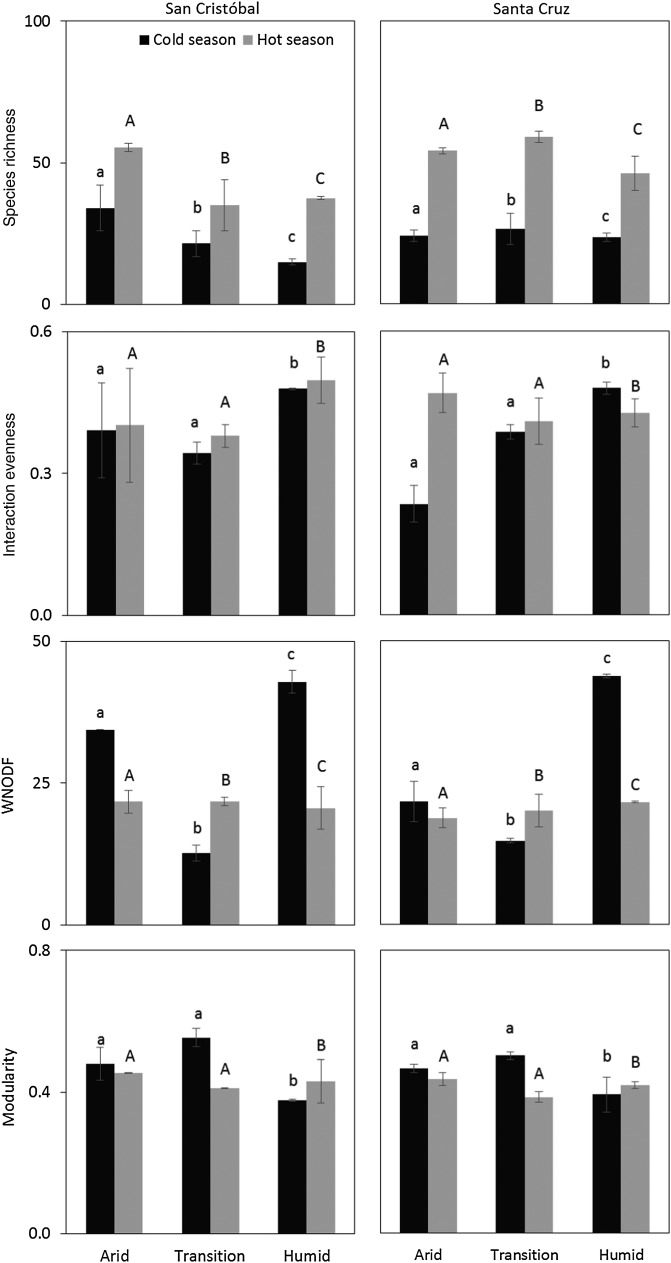
The mean (±1 SE) of the network parameters for each island, habitat and season. Data are from 2010. Only parameters that showed significant differences are shown. For each island and season, bars with the same letter indicate no differences across habitats (*P* > 0.05).

Data from 2010 showed that species richness was somewhat higher in Santa Cruz than in San Cristóbal (Table 2), although differences were not significant (*χ*^2^ = 18.26, d.f. = 3, *P* < 0.001) (Fig. 2). There were significant differences among habitats in the number of species in the network (*χ*^2^ = 27.17, d.f. = 4, *P* < 0.001), arid zone showing higher number than either the transition or the humid zone, which did not differ from each other (Fig. 2). A significant interaction between island and habitat was found (*χ*^2^ = 12.74, d.f. = 2, *P* < 0.01), as differences among habitats were not consistent between Santa Cruz and San Cristóbal (Fig. 2). On both islands, networks were larger in the hot/rainy season, when most flowers are in bloom and more insects are flying, than in the cold season (*χ*^2^ = 95.91, d.f. = 1, *P* < 0.001). The level of plant invasion showed no effect on species richness (*χ*^2^ = 0.52, d.f. = 1, *P* = 0.47) and was not included in the best model.

**Table 2.** Network-level parameters of the 36 matrices corresponding to the first year of the study and the 12 matrices built for the second year, in which only the hot season was considered. None of the modularity values (M) showed to be significant (all *P* values >0.05). P, number of plants; A, number of animals (pollinators); S, total number of species in the network; C, connectance; IE, interaction evenness; *H*′_2_, network specialization; IAc, corrected interaction asymmetry; WNODF, weighted nestedness (asterisks imply that it is significant); M, modularity; n_modules, number of modules in the network. **P ≤ 0.01, *P < 0.05.

Year	Island	Season	Habitat	Invasion level	P	A	S	C	IE	$H_{2}^{\prime }$	IAc	WNODF	M	n_modules
2010	San Cristóbal	Hot	Arid	7.59	28	29	57	0.12	0.28	0.82	0.01	23.66**	0.45	5
2010	San Cristóbal	Cold	Transition	72.75	11	15	26	0.17	0.37	0.89	0.12	11.25**	0.58	4
2010	San Cristóbal	Cold	Humid	0.65	6	8	14	0.35	0.48	0.18	0.07	44.88	0.37	4
2010	San Cristóbal	Cold	Humid	0.24	6	10	16	0.33	0.48	0.63	0.13	40.83**	0.38	3
2010	San Cristóbal	Hot	Arid	0.42	14	40	54	0.16	0.52	0.69	0.24	19.74**	0.45	5
2010	San Cristóbal	Hot	Transition	41.79	17	27	44	0.15	0.36	0.72	0.12	21.05**	0.41	7
2010	San Cristóbal	Hot	Transition	53.74	12	14	26	0.21	0.4	0.48	0.04	22.53**	0.41	6
2010	San Cristóbal	Hot	Humid	0.32	12	26	38	0.21	0.55	0.64	0.17	24.32**	0.37	5
2010	San Cristóbal	Hot	Humid	5.56	13	24	37	0.17	0.45	0.63	0.17	16.85**	0.49	5
2010	San Cristóbal	Cold	Arid	16.76	17	25	42	0.13	0.49	0.75	0.12	34.42*	0.53	6
2010	San Cristóbal	Cold	Arid	0	8	18	26	0.22	0.29	0.69	0.24	34.42*	0.43	5
2010	San Cristóbal	Cold	Transition	58.19	8	9	17	0.22	0.32	0.82	0.05	14.06**	0.53	5
2010	Santa Cruz	Hot	Arid	2.47	24	29	53	0.13	0.51	0.5	0.05	20.46**	0.42	6
2010	Santa Cruz	Cold	Transition	0.41	11	10	21	0.22	0.4	0.55	−0.03	14.27	0.51	6
2010	Santa Cruz	Cold	Humid	17.28	13	9	22	0.27	0.47	0.38	−0.09	44.15*	0.34	5
2010	Santa Cruz	Cold	Humid	8.16	15	10	25	0.21	0.49	0.38	−0.13	43.42	0.44	4
2010	Santa Cruz	Hot	Arid	0.69	24	31	55	0.12	0.43	0.71	0.07	17.06**	0.45	7
2010	Santa Cruz	Hot	Transition	61.91	20	37	57	0.15	0.36	0.67	0.12	17.2**	0.4	7
2010	Santa Cruz	Hot	Transition	18.08	22	39	61	0.16	0.46	0.68	0.1	22.86**	0.37	6
2010	Santa Cruz	Hot	Humid	16.81	14	26	40	0.18	0.46	0.66	0.14	21.44**	0.41	6
2010	Santa Cruz	Hot	Humid	27.01	21	31	52	0.14	0.4	0.71	0.09	21.72**	0.43	6
2010	Santa Cruz	Cold	Arid	0.01	8	14	22	0.23	0.2	0.72	0.15	18.15**	0.45	5
2010	Santa Cruz	Cold	Arid	0.01	9	17	26	0.2	0.27	0.2	0.19	25.15**	0.48	5
2010	Santa Cruz	Cold	Transition	11.68	9	23	32	0.22	0.37	0.56	0.24	15.16**	0.49	5
2011	San Cristóbal	Hot	Arid	8.25	15	19	34	0.13	0.48	0.69	0.09	9.55**	0.61	9
2011	San Cristóbal	Hot	Arid	2.29	10	22	32	0.17	0.37	0.31	0.27	14.01**	0.52	7
2011	San Cristóbal	Hot	Transition	16.99	13	18	31	0.13	0.4	0.74	0.14	14.79**	0.63	7
2011	San Cristóbal	Hot	Transition	61.91	11	13	24	0.17	0.31	0.87	0.07	14.29**	0.61	5
2011	San Cristóbal	Hot	Humid	4.56	5	4	9	0.4	0.34	0.42	−0.08	56.25*	0.39	3
2011	San Cristóbal	Hot	Humid	1.03	10	14	24	0.19	0.52	0.58	0.12	19.49	0.5	6
2011	Santa Cruz	Hot	Arid	2.35	25	67	92	0.09	0.47	0.52	0.24	16.3**	0.49	6
2011	Santa Cruz	Hot	Arid	1.73	24	43	67	0.13	0.45	0.58	0.12	18.98**	0.39	6
2011	Santa Cruz	Hot	Transition	17.48	13	41	54	0.13	0.41	0.56	0.33	15.19**	0.53	7
2011	Santa Cruz	Hot	Transition	52.41	19	47	66	0.14	0.45	0.6	0.18	25.06**	0.38	5
2011	Santa Cruz	Hot	Humid	43.28	11	25	36	0.19	0.43	0.74	0.22	18.06**	0.46	5
2011	Santa Cruz	Hot	Humid	71.93	22	35	57	0.14	0.44	0.62	0.1	17.09**	0.41	5

**Figure 3.**
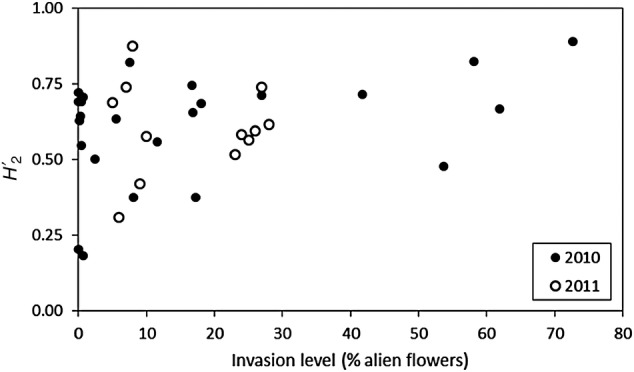
Relationship between the level of invasion (i.e. fraction of alien flowers out of all flowers in the site) and the level of network specialization *H*′_2_ found during the 2 years of the study. Data from the two islands and the three habitats are pooled. The association is marginally significant in the two cases (*t* = 1.9, *P* = 0.07 and *t* = 2.14, *P* = 0.06, in 2010 and 2011, respectively).

The fraction of realized interactions out of all possible in the network (*connectance*) did not vary either between islands, habitats or seasons (Fig. 2), and it was not influenced by the level of invasion (all *P* > 0.05). The same result was found for *interaction asymmetry*, which indicates the difference in the dependence of animals on plants and *vice versa*. Interaction evenness, which measures the uniformity in the distribution of interaction frequencies differed only across habitats, i.e. habitat was the only factor included in the best model (*χ*^2^ = 0.05, d.f. = 2, *P* = 0.02). The humid zone showed a more even frequency of interactions than the arid and the transition zones (Fig. 2). Interaction evenness was also independent of invasion level (*χ*^2^ = 1.90, d.f. = 1, *P* = 0.17). In contrast, the best model for *complementary specialization* $\left(H_{2}^{\prime }\right)$ included only invasion level (*χ*^2^ = 0.11, d.f. = 1, *P* = 0.057); a high fraction of alien flowers in the community was positively associated with higher $H_{2}^{\prime }$ (Fig. 3), i.e. with higher levels of selectiveness or niche differentiation, implying that species tended to visit (pollinators) or be visited (plants) by partners more frequently than expected from the relative abundances of the latter.

Habitat and season were the only variables included in the best model predicting *quantitative nestedness* (*WNODF*). Networks from the humid zone showed significantly higher levels of nestedness than those from the transition or the arid zone and (*χ*^2^ = 890.5, d.f. = 2, *P* < 0.001) (Table 2 and Fig. 2). Nestedness was higher in the cold than in the hot season (*χ*^2^ = 347.1, d.f. = 1, *P* = 0.01) (Fig. 2). The level of invasion did not affect the nested pattern of the networks, which was significant in 30 out of the 36 networks (Table 2). Finally, despite none of the networks was significantly modular (i.e. when compared to a null model), the degree of modularity (*M*) was slightly lower in the humid zone than in either the arid or the transition zone (*χ*^2^ = 0.02, d.f. = 2, *P* = 0.04) and was marginally higher in the cold than in the hot *season* (*χ*^2^ = 0.01, d.f. = 1, *P* = 0.07) (Fig. 2).

In 2011, the number of species in the networks was almost twice as high in Santa Cruz as in San Cristóbal (*χ*^2^ = 93.13, d.f. = 1, *P* < 0.001). Again, the humid zone showed the lowest species richness (*χ*^2^ = 28.31, d.f. = 2, *P* < 0.001). This year we found no significant differences in interaction evenness, nestedness or modularity across habitats **[see Supporting Information—Fig. S2]**.

### Spatio-temporal patterns at the species level

In 2010, pollinators in Santa Cruz had a higher *linkage level* (*L_a_*) than in San Cristóbal (*χ*^2^ = 6.13, d.f. = 1, *P* = 0.01) and also tended to visit more plant species in the humid than in the arid zone (*χ*^2^ = 9.11, d.f. = 4, *P* =0.05), and more in the hot than in the cold season (*χ*^2^ = 20.98, d.f. = 3, *P* < 0.001). There was a significant interaction between habitat and season (*χ*^2^ = 9.11, d.f. = 2, *P* = 0.01), as differences among habitats were not consistent between the two seasons **[see Supporting Information—Fig. S3]**. Results were consistent in 2011, except that this year *L_a_* was positively influenced by invasion level (*χ*^2^ = 10.89, d.f. = 1, *P* < 0.001); pollinators interacted with more plant species in sites with a greater fraction of alien flowers. There was a significant interaction between island and habitat, as differences among habitats varied slightly between the two islands **[see Supporting Information—Fig. S3]**. The other two parameters at the pollinator species level, *specialization level* (*d*′) and *strength* (*st*), could not be predicted by any of the variables included in the models, i.e. they did not differ between islands, habitats, or seasons and were not influenced by invasion levels.

Regarding *plant linkage level* (*L_p_*), the transition zone showed higher values than either the arid or the humid zone in 2010 (*χ*^2^ = 16.39, d.f. = 6, *P* = 0.01); differences among habitats were more marked in San Cristóbal than in Santa Cruz (*χ*^2^ = 13.24, d.f.= 2, *P* = 0.001) **[see Supporting Information—Fig. S3]**. In 2011, *L_p_* was higher in Santa Cruz than in San Cristóbal (*χ*^2^ = 29.35, d.f. = 1, *P* < 0.001), and it was lower in the humid zone than in the other two habitats (*χ*^2^ = 6.04, d.f. = 2, *P* < 0.05). In contrast, neither *d*′ nor *st* were significantly influenced by any of the predictor variables included in any of the models.

### Differences between alien and native species in interaction patterns

**Figure 4.**
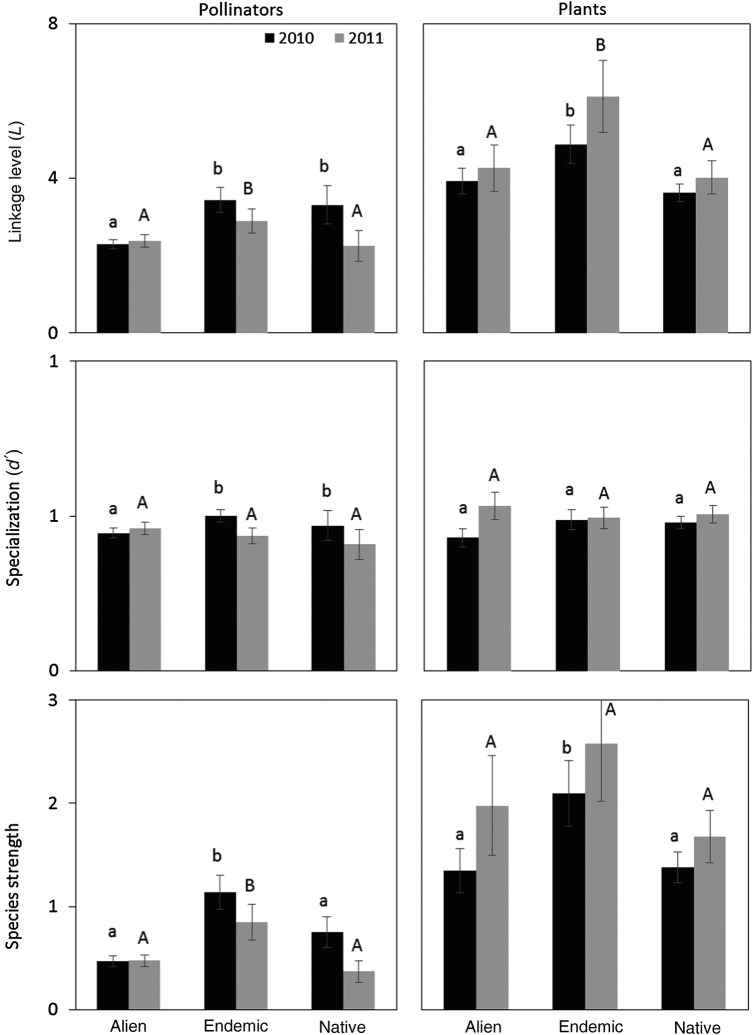
Mean (±1 SE) of the species-level parameters analysed in this study, for both pollinators and plants, showing differences among species of different origin for the 2 years of the study. Data from the two islands, three habitats and two seasons were pooled here for simplification. For each year, bars with the same letter indicate no differences across habitats (*P* > 0.05).

In both years, alien pollinators showed lower linkage levels than both endemic and non-endemic natives (*z* = 6.45, d.f. = 415 and *z* = 3.83, d.f. = 265, *P* < 0.001, respectively), although in 2011 aliens and non-endemic natives did not differ significantly (*z* = 0.40, d.f. = 265, *P* = 0.69) (Fig. 4). Alien pollinators had lower *d*′ and *st* values than endemic ones in 2010 (*t* = 2.05, d.f. = 415, *P* = 0.04 and *t* = 4.74, d.f. = 415, *P* < 0.001, respectively; Fig. 4). In 2011, *d*′ did not differ between the two groups but *st* was again significantly lower for alien than for endemic pollinators (*t* = 2.56, d.f. = 265, *P* = 0.01; Fig. 4).

On the other hand, in both years, alien plants showed lower *L_p_* and *st* than endemic plants, whereas they did not differ significantly from non-endemic native species. No differences were found in *d*′ depending upon plant species' origin (Fig. 4).

## Discussion

### Spatio-temporal network patterns and influence of plant invasion

Despite network size being larger in Santa Cruz than in San Cristóbal, especially in 2011 when it was twice as large, the overall pollination network structure was similar between the two islands. Strong spatial variation in network structure was detected, however, across habitats. The arid zone, which includes the vast majority of the land area and bears the highest species richness, supported the largest pollination networks. In contrast, the transition and the humid zone were more similar in size, though this was not consistent between islands or years. Flower and insect abundance are known to be influenced by abiotic conditions such as temperature or rainfall which can vary much spatially and temporally (Ziegler 1995; Trueman and d'Ozouville 2010). Alien plant species represented up to 40 % of the plants in some networks, particularly in the humid zone of Santa Cruz and in the transition zone in San Cristóbal. However, overall network size was not affected by the level of invasion—measured as the proportion of alien flowers—suggesting both that alien plants do not differ from natives with respect to the diversity of their pollinators and that aliens do not displace native plant species in the pollination networks. Habitats also differed in interaction evenness, nestedness and modularity. The uniformity in the distribution of interaction frequencies was higher in the humid habitat than in the two other habitats. In a previous study in the Galápagos (Traveset *et al*. 2013), a decrease in interaction evenness was observed along a gradient of invasion intensity at the island scale, being attributed to shifts in the proportion of strong and weak interactions in the network. However, the present work showed no effect of invasion level on this network parameter and, actually, the humid habitat is that bearing the highest fraction of alien species. Interaction evenness has been reported to increase after an invasion in one study on seed dispersal networks (Heleno *et al*. 2013*b*) but not in another (Heleno *et al*. 2013*a*). Hence, further data are needed to generalize about how this network parameter, known to be inversely related to network stability (Rooney and McCann 2012), is influenced by alien invasions. The humid habitat showed the strongest nested pattern (in which specialist species link to a subset of species with which generalists also interact), which could also be attributed, at least partly, to its high level of invasion. The degree of nestedness has been found to increase with the integration of alien species (Padrón *et al*. 2009; Santos *et al*. 2012); this is because aliens tend to be generalist species and/or are linked to generalist species (Aizen *et al*. 2008; Traveset *et al*. 2013). Thus, although the level of invasion overall was a poor predictor of nestedness, we cannot discard the possibility that a higher incidence of alien flowers enhances a nested pattern in a habitat. Modularity—another common parameter that informs us on how cohesive the network is and how vulnerable it can be to different types of disturbances (Olesen *et al*. 2007)—was lower in the humid zone, i.e. this zone had a weaker segregation of species into cores of strong interactions, than the arid and transition zones. The lower modularity in the humid zone might be associated with its lower plant and animal diversity compared with the transition and arid zones, and also with its relatively higher linkage levels (see below). The level of invasion has been documented to decrease modularity, and thus to enhance network cohesiveness in some studies (Santos *et al*. 2012; Albrecht *et al*. 2014). It is thus possible that the lower modularity in the humid habitat is partly due to its higher incidence of aliens. A low modularity has potential effects on network functioning, reciprocal selection regimes and the cascade of perturbations throughout the network (Albrecht *et al*. 2014).

Other network descriptors, such as connectance, interaction strength asymmetry and network complementary specialization $(H_{2}^{\prime }),$ did not vary much either in space or time. The level of network connectance, which is inversely related to network size, was both spatially and temporally consistent, despite species richness in each network varying across islands, habitats and seasons. This parameter is a measure, albeit crude, of network generalization level and, as expected from other island studies (Olesen and Jordano 2002; Traveset *et al*. 2013), we found relatively high values (∼18 % on average, ranging from 12 to 40 %, across the 36 matrices analysed). No effect of invasion level on connectance was observed, which is consistent with previous studies (Forup and Memmott 2005; Heleno *et al*. 2012), although network rewiring can actually occur and, as a result, the number of interactions between native species can decrease (Aizen *et al*. 2008; Padrón *et al*. 2009; Kaiser-Bunbury *et al*. 2011). Besides being consistent in space and time, most values of interaction strength asymmetry were positive which indicates that animals are more dependent upon plants than *vice versa* (Blüthgen *et al*. 2007), a result commonly found in other oceanic archipelagos (Kaiser-Bunbury *et al*. 2010; Traveset *et al*. 2015*b*), and a pattern not found to be influenced by invasion level in this study. Finally, an interesting finding from our study was that $H_{2}^{\prime }$ increased with the level of plant invasion, implying that species become more selective in their choice of mutualists by being compelled to interact with less abundant partners as invasion progresses. This finding contrasts with results from other studies which have reported a decrease in $H_{2}^{\prime }$ after an invasion (Heleno *et al*. 2013*b*).

Regarding the species-level parameters, pollinators tended to visit more plant species in Santa Cruz than in San Cristóbal, what can be attributed to the higher plant species richness in the former. Pollinators were also more generalist in the humid zone even though here the number of plants is lower than in the other two zones. It is possible, thus, that the lower amount of floral resources in the humid zone promotes insects visiting more plant species, as has been found in a number of island studies (Olesen *et al*. 2002; Kaiser-Bunbury *et al*. 2009; Padrón *et al*. 2009; Traveset *et al*. 2013). Interestingly, one of the years (2011), pollinators visited more plant species at sites with a greater fraction of alien flowers, suggesting that pollinators might be attracted to the new species which in turn would enhance their visitation to the other native plants in the community. Such ‘facilitative’ effects of alien plant species on native ones have been often reported in different systems (e.g. Moeller 2005; Jakobsson *et al*. 2009). Plant species, on the other hand, showed higher generalization levels in their pollination interactions in Santa Cruz than in San Cristóbal, at least in 2011 when more insect species were found on the former island. Plants were visited by less pollinator species in the humid zone, as the total number of pollinators is also lower in this zone compared with the other two zones. The other two parameters, species specialization *d*′ and strength *st*, were highly consistent in space. Both pollinators and plants had a similar level of selectiveness in their flower or pollinator use, respectively, and were also equally important to the plant or pollinator communities, respectively, in the two islands and in the three habitats. A fairly constant value of *d*′ for both plants and pollinators has been previously reported across five of the Galápagos Islands (Traveset *et al*. 2013). Moreover, those two parameters were not influenced by plant invasion level. In contrast, at least one study (of seed-dispersal networks) has reported the level of invasion to decrease species specialization *d*′ of native species (Heleno *et al*. 2013*a*).

Except for a few differences between pollination networks of different habitats, our results were highly consistent between the 2 years of the study, which were both considered ‘normal’ years in terms of precipitation and sea surface patterns (FCD Weather report, data not shown), despite the usual fluctuations in flower production and flower-visitors' presence/abundance. Thus, we focus on the temporal differences observed between seasons. All pollination networks were larger during the hot rainy season, when more plant species are blooming and more insects are flying, than in the cold dry season. Both pollinators and plants actually showed higher linkage levels in the hot than in the cold season, given the greater availability of partner species in the former. Moreover, networks were more nested in the cold than the hot season after controlling for network size, which influences this parameter. Such temporal difference in the degree of nestedness suggests that the interactions in the hot season tend to be more specific, with specialist species interacting more than expected with each other and less so with generalists.

### Integration of alien species on pollination networks

Alien pollinators were consistently found to visit fewer plant species than endemic pollinators and, at least one year, also visited fewer plant species than native pollinators, which suggests that these newly-arrived species are focusing flower visitation on species with particular traits. However, the fact that alien pollinators also showed lower levels of selectiveness than endemic pollinators implies that they tend to visit more abundant flower resources compared with endemic pollinators, which visit even rare flowers. Likewise, species strength was consistently lower for alien than endemic pollinators, indicating that the former are less important to plants. In a previous study focusing on the pollination networks of the arid zone in five Galápagos islands, we found that alien insects had more links than either endemics or non-endemic natives (Traveset *et al*. 2013), which suggests that the inclusion of the two other habitats, transition and humid, in the present study masks that pattern and/or that Santa Cruz and San Cristóbal are somewhat outliers in archipelago wide patterns, possibly due to the high level of disturbance.

Alien plants were also consistently more specialized than endemic plants, although they were similar to native species. In contrast to pollinators, plants showed similar selectiveness regardless of their origin, but again, endemic plant species were more important to the pollinator community than alien plants. These findings were consistent with our previous study (Traveset *et al*. 2013). It might be possible that aliens do not rely as much on pollinators as native species do. However, no data are currently available on the breeding system for the large majority of plants and, thus, future studies are needed to test this hypothesis.

## Conclusions

The structure of pollination networks is highly consistent on the two most disturbed islands of the Galápagos archipelago. Differences in network structure exist across the main habitats. The most widespread arid habitat consistently bears the largest pollination networks and differs strongly from the humid habitat in descriptors such as interaction evenness, nestedness and modularity. The transition habitat between the arid and the humid zone shows pollination networks more similar in structure to those in the arid than in the humid areas. The humid habitat is also the most invaded by alien species and this could partly explain some of the differences in its network structure, such as its more nested pattern and its lower modularity level compared with the arid and the transition zones. Pollinators appear to interact with more plants in the humid habitat than in the arid one. The incidence of alien flowers might actually increase the level of pollinator generalization, although results are inconclusive as this was observed in only one of the two study years. Overall, the level of invasion has a weak influence on pollination network structure and seems to be associated with only one metric, $H_{2}^{\prime }$ which measures the level of selectiveness; thus, as invasion progresses, species in the network appear to become more selective in their choice of partners, interacting with less abundant species more than would be expected by chance.

Pollination networks are larger during the hot/rainy season, when most flowers are in bloom and more insects are flying, than in the cold/dry season. They are also more nested in the hot season, and thus probably more robust to disturbances. Pollinators visit more plant species, and plants are visited by more pollinator species, during the hot season. In the cold season, the number of insects is especially low in the humid zones and thus the number of pollinators visiting plants is also lower in that season and habitat. In contrast, both pollinator and plant selectiveness (*d*′) and strength (*st*, importance to the plant and pollinator community, respectively) were spatially and temporally consistent and not influenced by alien plants.

Alien pollinators interacted with fewer plants, were less selective in their choice (i.e. tended to visit the most abundant species) and were less important to the plant community (i.e. showed lower species strength) than endemic and native pollinators. They, however, infiltrated the native communities of all habitats and in both seasons and currently represent over 40 % of all recorded pollination interactions. Alien plants, on the other hand, were visited by approximately the same number of pollinators as natives—but less than endemic plants—implying that they are also well integrated into the native communities. In this study, we found a rather feeble effect of alien plants on the structure of pollination networks. As previously mentioned in the methods, our study intentionally considered sites that are not completely disturbed by highly invasive species (e.g. *Psidium guava*, *Rubus nivaeus*, *Syzygium jambos*) which have displaced many native species in the invaded areas, mainly in the humid zones (Guézou *et al*. 2010). Hence, the overall weak effect we found does not imply a weak influence of plant invasions on the reproductive success of native species. The fact that alien plant species are present in all habitats and in both seasons and that they are involved in ∼25 % of all pollination interactions, actually leads us to think that their effect on the functioning of native communities is far from negligible.

## Sources of Funding

This research is part of a larger project funded by BBVA Foundation and is also framed within project CGL-2013-44386-P financed by the Spanish Government. R.H. was supported by the Portuguese grant FCT-IF/00441/2013, and by the Marie Curie action FP7-2012-CIG-321794.

## Contributions by the Authors

A.T., J.M.O. and R.H. designed the study, S.C. collected the data and did some preliminary analyses, and A.T. and R.H. performed the final analyses. A.T. led the writing and all authors contributed to the text. All authors read and approved the final manuscript.

## Conflict of Interest Statement

None declared.

## Supplementary Material

Additional Information

## Supporting Information

The following additional information is available in the online version of this article –

**Appendix S1.** Definitions of the metrics used in this study to describe network structure.

**Table S1.** Complete list of flowering species observed in this study, including origin, overall number of observed visits per plant species and estimated flower abundance (calculated by multiplying the mean number of flowers on two individuals per transect by the total number of individuals counted along the transects). The eight plant species that have not received any recorded visit during the censuses are marked with an asterisk.

**Table S2.** Complete list of flower-visitors found in this study, including origin and the overall number of observed visits per animal species.

**Figure S1.** Illustration of the entire pollination network, comprising data from the two islands (Santa Cruz and San Cristóbal), the three habitats (arid, transition and humid zones) and the two seasons (hot and cold). Data from 2010 to 2011 are also pooled. Plant species are depicted at the bottom of the network, whereas pollinators are at the top. Alien (A) plants and their links are represented in red to illustrate the magnitude of the interactions in which they are involved, whereas endemic (Nze) and non-endemic natives (Nt) are represented in black and grey, respectively. Questionably native plants (Nq) are also shown in grey.

**Figure S2.** Mean (±1 SE) of network metrics showing differences between the two seasons across habitats for each study island in the hot season of 2011. Data on IE are shown for comparison with data from Fig. 2 (2010 data), although differences across habitats were not significant this year. For each island, bars with the same letters indicate no differences across habitats (*P* > 0.05).

**Figure S3.** Mean (±1 SD) of the species-level parameters analysed in this study, for both pollinators and plants, showing differences among species from different habitats for the two islands, and the two seasons of 2010. Bars with the same letters on each graph indicate no differences across habitats (*P* > 0.05).
