# Supplementary material for: Space, time and aliens: charting the dynamic structure of Galápagos pollination networks
Source: AoB Plants. 2015 Jun 23;7:plv068. doi: 10.1093/aobpla/plv068 (PMC4522039; doi:10.1093/aobpla/plv068)
Supplement: Additional Information [file supp_7_plv068_index.html]

Space, time and aliens: charting the dynamic structure of Galápagos pollination networks — Additional Information 

# Space, time and aliens: charting the dynamic structure of Galápagos pollination networks

## Additional Information

Additional Information

- Additional Information - Docx file
