# Supplementary material for: Space, time and aliens: charting the dynamic structure of Galápagos pollination networks
Source: AoB Plants. 2015 Jun 23;7:plv068. doi: 10.1093/aobpla/plv068 (PMC4522039; doi:10.1093/aobpla/plv068)

**Supporting Information Files**

**Appendix S1** Definitions of the metrics used in this study to describe network structure.

**Network level parameters:**

1. *Species richness*. Total number of plants and animals in the bipartite network.
2. *Connectance*. Realized proportion of possible links: sum of links divided by number of cells in the matrix (the latter being the product between the number of higher trophic level species –animals, in our case- and the number of lower trophic level species –plants, in our case).
3. *Interaction asymmetry (or interaction strength asymmetry)*. Difference between the interaction strength (i.e. the relative frequency) of each animal species *i* on each plant species *j* and its reverse from the plant perspective, standardized by the sum of interaction strength values of species *i* on *j* and of species *j* on *i* (Bascompte *et al*. 2006; extended by Blüthgen 2010). Values vary between −1 and 1, where positive values indicate a high dependence of animal on plant species and negative values indicate the opposite. Given that this variable, by its mathematical definition, is closely associated with web asymmetry, this correlation is accounted with null models (see further details in Blüthgen 2010). Thus, for each network in the data set, we computed 1000 randomized interaction matrices simulated with the Patefield algorithm, which randomly redistributes interaction events among all cells of the matrix while holding the number of interaction events per species constant. Thus, web asymmetries were held constant in all simulated networks, while interactions were reallocated between pairs of species according to species interaction frequencies. The difference between observed asymmetries of interaction strength and the mean asymmetry of interaction strength across the 1000 simulations gives the null-model-corrected asymmetry of interaction strength.
4. *Interaction evenness* (*IE*), a measure of the uniformity of interactions between species in a network, based on Shannon’s evenness (Tylianakis *et al*. 2007). An uneven network has a high skewness in the distribution of interaction weights. It ranges from 0 (completely uneven) to 1 (completely uniform).
5. *Network specialization* *H_2_'* (Blüthgen *et al*. 2006). Measure of the level of network selectiveness, is also derived from Shannon entropy and is related to weighted specialization (*d*’) across all species. It ranges between 0 (opportunistic, high niche overlap) and 1 (selective, high niche differentiation).
6. *Weighted NODF* (WNODF; Weighted Nestedness based on Overlap and Decreasing Fill; Almeida-Neto & Ulrich 2011), a measure of the degree of nestedness for quantitative data. Networks are nested if those species with fewer interactions are preferentially associated with a subset of species that interact with the most connected ones. It ranges from 0 (not nested network) to 100 (highly nested network). The significance of WNODF was tested by comparing it with that obtained from 1000 randomized networks constrained by the total abundances of each row and column as the empirical one; we used the null model *rc,* which assigns individuals to matrix cells proportional to observed row and column abundance totals until, for each row and column, total abundances are reached.
7. *Modularity* (**M**), refers to the existence of subsets (modules) of more closely interacting species with relatively few or no interactions to other subsets (Guimerà *et al.* 2010). It was obtained with the software MODULAR (Marquitti *et al*. 2014).

**Species level parameters:**

1. *Species specialization for plants* ***(d_’p_)*** *and animals* ***(d_’a_***) (Blüthgen *et al*. 2006) gives levels of specialization of each species, accounting for the available resources provided by the interaction partners (calculated as marginal totals in the matrix). This index increases with the deviation from random selection of the available interaction partners based on their abundance. Thus, a pollinator species, for example, that visits flowering plant species proportionally to their availability in the community is considered generalized, while a species that visits rare plants disproportionately is considered specialized. It is actually an index of selectivity, thus we could call the ‘generalized’ species as ‘opportunistic’ and the ‘specialized’ species as ‘selective’. Note that a particular species can be opportunistic in one community but selective in another one, depending on the abundance of its partners.
2. *Species strength.* The strength of an animal species (***st’_a_***) is defined as the sum of dependencies of the plants relying on the particular pollinator, whereas the strength of a plant (***st’_p_***) is the sum of dependencies of the pollinators relying on the particular plant species (Bascompte *et al*. 2006). We calculated the dependence of a pollinator species on a particular plant species by dividing the number of visits of that pollinator to that plant by the total number of pollinator visits that plant receives. Conversely, the dependence of a plant species on a particular pollinator species is obtained by dividing the number of times the pollinator visited the plant by the total number of visits of that pollinator species to plants in the community.

**References**

Almeida-Neto M., Ulrich W. 2011. A straightforward computational approach for measuring nestedness using quantitative matrices. *Environmental Modelling & Software*, **26**: 173–178.

Bascompte J, Jordano P, Olesen JM. 2006. Asymmetric coevolutionary networks facilitate biodiversity maintenance. *Science,* **312:** 431–433.

Blüthgen N, Menzel F, Blüthgen N. 2006 Measuring specialization in species interaction networks. *BMC Ecology* **6:** 12.

Blüthgen N. 2010. Why network analysis is often disconnected from community ecology: A critique and an ecologist’s guide. *Basic and Applied Ecology*, **11:** 185–195.

Guimerà R, Amaral LAN. 2005. Functional cartography of complex metabolic networks. *Nature*, **433:** 895–900.

Guimerà R, Stouffer DB, Sales-Pardo M., Leicht EA, Newman MEJ, Amaral LAN. 2010. Origin of compartmentalization in food webs. *Ecology,* **91:** 2941–2951.

Marquitti FD, Guimaraes PR Jr , Pires MM, Bittencourt LF. 2014. MODULAR: software for the autonomous computation of modularity in large network sets*. Ecography,* **37**: 221–224.

Tylianakis JM, Tscharntke T, Lewis OT. 2007. Habitat modification alters the structure of tropical host-parasitoid food webs. *Nature*, **445**: 202–205.

**Table S1** Complete list of flowering species observed in this study, including origin, overall number of observed visits per plat species and estimated flower abundance (calculated by multiplying the mean number of flowers on two individuals per transect by the total number of individuals counted along the transects). The eight plant species that have not received any recorded visit during the censuses are marked with an asterisk.

| **Family** | **Species** | **Origin** | **Total observed number of visits** | **Total estimated flower abundance** |
| --- | --- | --- | --- | --- |
| Acanthaceae | Blechum pyramidatum | Native | 7 | 939 |
| Acanthaceae | Justicia galapagana | Endemic | 11 | 246 |
| Amaranthaceae | Alternanthera echinocephala | Native | 93 | 162387 |
| Apocynaceae | Catharanthus roseus | Introduced | 10 | 666 |
| Apocynaceae | Vallesia glabra | Native | 42 | 3536 |
| Asteraceae | Acmella sodiroi | Introduced | 60 | 1756 |
| Asteraceae | Adenostemma platyphyllum | Introduced | 72 | 55249 |
| Asteraceae | Ageratum conyzoides | Native | 98 | 914977 |
| Asteraceae | Bidens pilosa | Questionable Native | 564 | 281940 |
| Asteraceae | Blainvillea dichotoma | Native | 107 | 549070 |
| Asteraceae | Centraterum punctatum* | Introduced | 0 | 28 |
| Asteraceae | Conyza canadensis | Introduced | 2 | 3000 |
| Asteraceae | Eclipta prostrata | Native | 17 | 623 |
| Asteraceae | Jaegeria gracilis | Endemic | 339 | 169159 |
| Asteraceae | Macraea laricifolia | Endemic | 53 | 60330 |
| Asteraceae | Porophyllum ruderale | Introduced | 55 | 5855 |
| Asteraceae | Pseudelephantopus spiralis | Introduced | 135 | 60906 |
| Asteraceae | Scalesia pedunculata | Endemic | 292 | 12505 |
| Asteraceae | Sonchus oleraceus* | Introduced | 0 | 23 |
| Asteraceae | Synedrella nodiflora | Introduced | 1 | 3362 |
| Boraginaceae | Cordia leucophlyctis | Endemic | 435 | 156645 |
| Boraginaceae | Cordia lutea | Native | 300 | 108473 |
| Boraginaceae | Heliotropium angiospermum | Native | 164 | 68879 |
| Boraginaceae | Tournefortia psilostachya | Native | 10 | 2838 |
| Boraginaceae | Tournefortia pubescens | Endemic | 137 | 285522 |
| Boraginaceae | Tournefortia rufo-sericea | Endemic | 221 | 139789 |
| Brassicaceae | Lepidium virginicum | Introduced | 12 | 4900 |
| Burseraceae | Bursera graveolens | Native | 8 | 23700 |
| Cactaceae | Opuntia echios | Endemic | 76 | 967 |
| Caesalpinaceae | Parkinsonia aculeata | Native | 156 | 9339 |
| Caesalpinaceae | Senna obtusifolia | Introduced | 3 | 830 |
| Caesalpinaceae | Senna occidentalis | Native | 25 | 1074 |
| Caryophyllaceae | Drymaria cordata | Native | 21 | 27924 |
| Celastraceae | Maytenus octogona | Native | 1162 | 140110 |
| Commelinaceae | Commelina diffusa | Native | 24 | 3066 |
| Commelinaceae | Tradescantia fluminensis | Introduced | 2 | 56 |
| Convolvulaceae | Evolvulus convolvuloides | Native | 11 | 48341 |
| Convolvulaceae | Evolvulus simplex | Native | 2 | 816 |
| Convolvulaceae | Ipomoea nil | Introduced | 11 | 1374 |
| Convolvulaceae | Ipomoea triloba | Native | 106 | 17911 |
| Convolvulaceae | Merremia aegyptia | Native | 31 | 24081 |
| Cucurbitaceae | Cucumis dipsaceus | Introduced | 11 | 569 |
| Cucurbitaceae | Momordica charantia | Introduced | 93 | 16371 |
| Euphorbiaceae | Croton scouleri | Endemic | 378 | 250401 |
| Euphorbiaceae | Hippomane mancinella | Native | 27 | 64125 |
| Fabaceae | Crotalaria pumila* | Native | 0 | 7 |
| Fabaceae | Crotalaria retusa | Introduced | 3 | 377 |
| Fabaceae | Desmodium glabrum | Questionable Native | 2 | 1460 |
| Fabaceae | Desmodium incanum | Questionable Native | 14 | 1580 |
| Fabaceae | Desmodium intortum | Questionable Native | 27 | 8016 |
| Fabaceae | Fabaceae | ? | 1 | 1104 |
| Fabaceae | Galactia striata | Native | 83 | 3042 |
| Fabaceae | Rhynchosia minima | Native | 271 | 58611 |
| Fabaceae | Tephrosia cinerea | Native | 35 | 844 |
| Fabaceae | Vigna luteola | Native | 7 | 115 |
| Hypoxidaceae | Hypoxis decumbens | Native | 20 | 534 |
| Lamiaceae | Hyptis pectinata | Introduced | 605 | 700414 |
| Lamiaceae | Hyptis rhomboidea | Introduced | 9 | 7158 |
| Lamiaceae | Hyptis sidifolia | Introduced | 4 | 8104 |
| Lamiaceae | Salvia occidentalis | Native | 15 | 6156 |
| Loasaceae | Mentzelia aspera | Native | 74 | 152479 |
| Lythraceae | Cuphea carthagenensis | Native | 143 | 56664 |
| Lythraceae | Cuphea racemosa | Introduced | 26 | 11802 |
| Malvaceae | Abutilon depauperatum | Endemic | 81 | 6365 |
| Malvaceae | Anoda acerifolia | Introduced | 75 | 2712 |
| Malvaceae | Bastardia viscosa | Native | 72 | 34634 |
| Malvaceae | Gossypium darwinii | Endemic | 24 | 452 |
| Malvaceae | Sida ciliaris | Introduced | 8 | 2753 |
| Malvaceae | Sida rhombifolia | Introduced | 145 | 65519 |
| Malvaceae | Sida salviifolia | Native | 38 | 7786 |
| Melastomataceae | Miconia robinsoniana | Endemic | 332 | 506955 |
| Mimosaceae | Acacia rorudiana | Questionable Endemic | 26 | 17737 |
| Mimosaceae | Desmanthus virgatus | Native | 14 | 3995 |
| Myrtaceae | Psidium galapageium* | Endemic | 0 | 121 |
| Myrtaceae | Psidium guajava | Introduced | 29 | 335 |
| Nyctaginaceae | Boerhavia caribaea | Native | 57 | 10248 |
| Nyctaginaceae | Boerhavia erecta | Native | 6 | 85 |
| Nyctaginaceae | Commicarpus tuberosus | Native | 107 | 30748 |
| Nyctaginaceae | Cryptocarpus pyriformis | Native | 235 | 1438738 |
| Nyctaginaceae | Pisonia floribunda* | Endemic | 0 | 300 |
| Onagraceae | Ludwigia leptocarpa | Native | 66 | 1571 |
| Oxalidaceae | Oxalis corniculata* | Introduced | 0 | 4 |
| Oxalidaceae | Oxalis corymbosa* | Introduced | 0 | 4 |
| Oxalidaceae | Oxalis dombeyi | Native | 4 | 316 |
| Passifloraceae | Passiflora edulis | Introduced | 78 | 315 |
| Passifloraceae | Passiflora foetida | Endemic | 48 | 3020 |
| Passifloraceae | Passiflora suberosa | Native | 11 | 95 |
| Plumbaginaceae | Plumbago scandens | Native | 141 | 22698 |
| Poaceae | Paspalum conjugatum | Questionable Native | 86 | 274362 |
| Polygonaceae | Polygonum galapagense | Endemic | 66 | 55596 |
| Portulacaceae | Portulaca oleracea | Questionable Native | 27 | 4572 |
| Portulacaceae | Talinum paniculatum | Introduced | 25 | 13470 |
| Rhamnaceae | Scutia spicata | Questionable Endemic | 90 | 69560 |
| Rosaceae | Rubus niveus | Introduced | 105 | 22552 |
| Rubiaceae | Chiococca alba | Native | 36 | 108328 |
| Rubiaceae | Diodia radula | Native | 376 | 175280 |
| Rubiaceae | Psychotria rufipes | Endemic | 63 | 6341 |
| Rutaceae | Citrus x aurantium | Introduced | 36 | 1100 |
| Rutaceae | Zanthoxylum fagara | Native | 8 | 1000 |
| Sapindaceae | Cardiospermum galapageium | Endemic | 115 | 27422 |
| Scrophulariaceae | Calceolaria meistantha | Native | 2 | 375 |
| Scrophulariaceae | Capraria biflora | Native | 12 | 660 |
| Solanaceae | Browallia americana | Introduced | 35 | 1928 |
| Solanaceae | Capsicum frutescens | Introduced | 4 | 22 |
| Solanaceae | Physalis angulata | Native | 7 | 301 |
| Solanaceae | Physalis pubescens | Native | 3 | 6 |
| Solanaceae | Solanum americanum* | Questionable Native | 0 | 447 |
| Solanaceae | Solanum pimpinellifolium | Introduced | 20 | 3336 |
| Sterculiaceae | Waltheria ovata | Native | 632 | 241892 |
| Valerianaceae | Valeriana chaerophylloides | Questionable Native | 64 | 4895 |
| Verbenaceae | Citharexylum gentryi | Introduced | 5 | 376 |
| Verbenaceae | Clerodendrum molle | Native | 230 | 44447 |
| Verbenaceae | Lantana camara | Introduced | 127 | 298359 |
| Verbenaceae | Lantana peduncularis | Endemic | 95 | 60882 |
| Verbenaceae | Phyla stringulosa | Native | 108 | 209907 |
| Verbenaceae | Stachytarpheta cayennensis | Introduced | 179 | 132000 |
| Verbenaceae | Verbena litoralis | Questionable Native | 71 | 45981 |
| Verbenaceae | Verbena sedula | Endemic | 60 | 28377 |
| Zygophyllaceae | Tribulus cistoides | Questionable Native | 122 | 3177 |

**Table S2** Complete list of flower visitors found in this study, including origin and the overall number of observed visits per animal species.

| **Class** | **Order** | **Species** | **Origin** | **Total observed visits** |
| --- | --- | --- | --- | --- |
| Aves | Passeriformes | Dendroica petechia | Endemic | 2 |
| Aves | Passeriformes | Geospiza fuliginosa | Endemic | 8 |
| Aves | Passeriformes | Geospiza scandens | Endemic | 3 |
| Entognatha | Collembola | Entomobryidae sp. 1 |  | 1 |
| Insecta | Coleoptera | Acanthoscelides fuscomaculatus | Endemic | 4 |
| Insecta | Coleoptera | Acanthoscelides manleyi | Native | 1 |
| Insecta | Coleoptera | Acanthoscelides rossi | Endemic | 15 |
| Insecta | Coleoptera | Amblycerus galapagoensis | Native | 1 |
| Insecta | Coleoptera | Brentus volvulus | Introduced | 1 |
| Insecta | Coleoptera | Calosoma sp. |  | 5 |
| Insecta | Coleoptera | Coccidophilus sp.1 |  | 5 |
| Insecta | Coleoptera | Coccinellidae sp. 1 |  | 1 |
| Insecta | Coleoptera | Coccinellidae sp. 2 |  | 1 |
| Insecta | Coleoptera | Conotelus sp. | Introduced | 5 |
| Insecta | Coleoptera | Cycloneda sanguinea | Native | 11 |
| Insecta | Coleoptera | Diomus anthony | Native | 1 |
| Insecta | Coleoptera | Dipropus puberulus | Native | 1 |
| Insecta | Coleoptera | Galapaganus ashlocki | Endemic | 1 |
| Insecta | Coleoptera | Galerucinae sp. 1 |  | 1 |
| Insecta | Coleoptera | Hypasclera collenetti | Endemic | 15 |
| Insecta | Coleoptera | Longitarsus galapagoensis | Endemic | 3 |
| Insecta | Coleoptera | Minographus sp. |  | 2 |
| Insecta | Coleoptera | Mordellistena galapagoensis | Endemic | 6 |
| Insecta | Coleoptera | Myochrous especies |  | 1 |
| Insecta | Coleoptera | Myochrous sp. | Questionable Native | 1 |
| Insecta | Coleoptera | Ormiscus variegatus | Endemic | 2 |
| Insecta | Coleoptera | Physorhinus galapagoensis | Endemic | 1 |
| Insecta | Coleoptera | Rodolia cardinalis | Introduced | 3 |
| Insecta | Coleoptera | Scutobruchus ceratioborus | Native | 2 |
| Insecta | Coleoptera | Sennius falcatus | Native | 3 |
| Insecta | Coleoptera | Thermonectus basillaris | Endemic | 3 |
| Insecta | Coleoptera | Xyleborus spinulosus | Questionable Native | 1 |
| Insecta | Diptera | Allograpta splendens | Endemic | 135 |
| Insecta | Diptera | Anastrepha fraterculus | Introduced | 13 |
| Insecta | Diptera | Asteiidae sp. 4 |  | 6 |
| Insecta | Diptera | Campiglossa crockeri | Endemic | 5 |
| Insecta | Diptera | Cecidomyiidae sp.1 |  | 2 |
| Insecta | Diptera | Chloropidae sp. |  | 6 |
| Insecta | Diptera | Chloropidae sp. 2 |  | 3 |
| Insecta | Diptera | Chrysanthrax primitivus | Endemic | 9 |
| Insecta | Diptera | Chrysomya albiceps | Introduced | 11 |
| Insecta | Diptera | Chrysomyia sp. 1 |  | 2 |
| Insecta | Diptera | Cochliomyia macellaria | Introduced | 5 |
| Insecta | Diptera | Cymoninus notabilis | Questionable Native | 4 |
| Insecta | Diptera | Cyrtoneuropsis rescita | Introduced | 1 |
| Insecta | Diptera | Dasyhelea mutabilis | Questionable Native | 30 |
| Insecta | Diptera | Dasyhelea sp. |  | 3 |
| Insecta | Diptera | Drosophila sp. |  | 4 |
| Insecta | Diptera | Drosophila sp. 1 |  | 3 |
| Insecta | Diptera | Drosophilidae sp.1 |  | 1 |
| Insecta | Diptera | Forcipomyia sp. 1 |  | 1 |
| Insecta | Diptera | Hyphantrophaga sp. | Introduced | 4 |
| Insecta | Diptera | Leia sp. |  | 3 |
| Insecta | Diptera | Lepidanthrax tinctus | Introduced | 7 |
| Insecta | Diptera | Limonia galapagoensis | Endemic | 42 |
| Insecta | Diptera | Limonia sp. 1 |  | 182 |
| Insecta | Diptera | Liohippelates sp. |  | 1 |
| Insecta | Diptera | Liohippelates sp.1 |  | 10 |
| Insecta | Diptera | Lonchaeidae sp. 1 |  | 1 |
| Insecta | Diptera | Lucilia pionia | Endemic | 3 |
| Insecta | Diptera | Lucilia sp. 1 |  | 27 |
| Insecta | Diptera | Mycetophilidae sp.1 |  | 6 |
| Insecta | Diptera | Nemotelus albiventris | Endemic | 3 |
| Insecta | Diptera | Neodexiopsis devia | Endemic | 1 |
| Insecta | Diptera | Ornidia obesa | Introduced | 54 |
| Insecta | Diptera | Oxysarcodexia taitensis | Introduced | 47 |
| Insecta | Diptera | Palaeosepsis armillata | Introduced | 6 |
| Insecta | Diptera | Palpada albifrons | Introduced | 78 |
| Insecta | Diptera | Philornis downsi | Introduced | 67 |
| Insecta | Diptera | Pieza sinclairi | Introduced | 2 |
| Insecta | Diptera | Pseudodoros clavatus | Introduced | 76 |
| Insecta | Diptera | Psychodidae sp. 1 |  | 1 |
| Insecta | Diptera | Sarcodexia lambens | Introduced | 41 |
| Insecta | Diptera | Sarcodexia sp. 1 |  | 4 |
| Insecta | Diptera | Sarcodexia sp. 2 |  | 15 |
| Insecta | Diptera | Sarcophagidae sp. 4 |  | 5 |
| Insecta | Diptera | Sarcophagidae sp. 5 |  | 12 |
| Insecta | Diptera | Scatopsidae sp. 1 |  | 2 |
| Insecta | Diptera | Sciara sp. |  | 8 |
| Insecta | Diptera | Sciaridae sp. 1 |  | 6 |
| Insecta | Diptera | Sciaridae sp. 2 |  | 36 |
| Insecta | Diptera | Sepsidae sp. 1 |  | 1 |
| Insecta | Diptera | Siphona sp. |  | 303 |
| Insecta | Diptera | Siphona sp.1 |  | 171 |
| Insecta | Diptera | Tachinidae sp. 2 |  | 2 |
| Insecta | Diptera | Tachinidae sp. 3 |  | 2 |
| Insecta | Diptera | Tachinidae sp. 4 |  | 1 |
| Insecta | Diptera | Tephritidae sp. 2 |  | 1 |
| Insecta | Diptera | Toxomerus crockeri | Endemic | 948 |
| Insecta | Diptera | Toxomerus politus | Introduced | 8 |
| Insecta | Diptera | Toxomerus sp.1 | Endemic | 11 |
| Insecta | Diptera | Tricharaea canuta | Introduced | 23 |
| Insecta | Diptera | Tricharaea occidua | Introduced | 100 |
| Insecta | Diptera | Volucellini sp. 1 |  | 1 |
| Insecta | Diptera | Xanthandrus agonis | Endemic | 3 |
| Insecta | Hemiptera | Alcaeorrhyncus sp. 1 | Introduced | 1 |
| Insecta | Hemiptera | Arhyssus sp. | Questionable Native | 3 |
| Insecta | Hemiptera | Arhyssus sp.1 |  | 5 |
| Insecta | Hemiptera | Engytatus modestus | Introduced | 6 |
| Insecta | Hemiptera | Horcias lacteiclavus |  | 3 |
| Insecta | Hemiptera | Metacanthus galapagensis | Endemic | 3 |
| Insecta | Hemiptera | Miridae sp. 3 |  | 10 |
| Insecta | Hemiptera | Nabis consimilis | Native | 4 |
| Insecta | Hemiptera | Nabis reductus | Endemic | 29 |
| Insecta | Hemiptera | Neortholomus usingeri | Endemic | 58 |
| Insecta | Hemiptera | Niesthrea sp. 1 |  | 3 |
| Insecta | Hemiptera | Nysius usitatus | Endemic | 11 |
| Insecta | Hemiptera | Pentatomidae sp.1 |  | 3 |
| Insecta | Hemiptera | Polymerus nigritulus | Endemic | 4 |
| Insecta | Hemiptera | Rhinacloa sp. 1 |  | 4 |
| Insecta | Hemiptera | Teleonemia prolixa | Native | 1 |
| Insecta | Hymenoptera | Anthidium vigintiduopunctatum | Introduced | 155 |
| Insecta | Hymenoptera | Apanteles sp. |  | 1 |
| Insecta | Hymenoptera | Asteiidae sp.1 |  | 4 |
| Insecta | Hymenoptera | Brachygastra lecheguana | Introduced | 546 |
| Insecta | Hymenoptera | Brachymyrmex heeri | Introduced | 313 |
| Insecta | Hymenoptera | Brachymyrmex sp | Introduced | 1 |
| Insecta | Hymenoptera | Braconidae sp.1 |  | 1 |
| Insecta | Hymenoptera | Bycertis sp. | Introduced | 73 |
| Insecta | Hymenoptera | Camponotus macilentus | Endemic | 1 |
| Insecta | Hymenoptera | Camponotus planus | Endemic | 20 |
| Insecta | Hymenoptera | Camponotus zonatus | Introduced | 91 |
| Insecta | Hymenoptera | Cardiocondyla emeryi | Introduced | 2 |
| Insecta | Hymenoptera | Cardiocondyla minutior | Introduced | 1 |
| Insecta | Hymenoptera | Cheloninae sp. 1 |  | 1 |
| Insecta | Hymenoptera | Cheloninae sp. 2 |  | 2 |
| Insecta | Hymenoptera | Cheloninae sp. 3 |  | 2 |
| Insecta | Hymenoptera | Chelonus sp. 1 |  | 2 |
| Insecta | Hymenoptera | Conura femorata | Introduced | 3 |
| Insecta | Hymenoptera | Enicospilus sp. |  | 48 |
| Insecta | Hymenoptera | Figitidae sp. 1 | Introduced | 1 |
| Insecta | Hymenoptera | Ichneumomidae |  | 1 |
| Insecta | Hymenoptera | Kapala sp. |  | 3 |
| Insecta | Hymenoptera | Kapala sp.1 |  | 1 |
| Insecta | Hymenoptera | Monomorium destructor | Introduced | 186 |
| Insecta | Hymenoptera | Monomorium floricola | Introduced | 127 |
| Insecta | Hymenoptera | Monomorium sp. | Introduced | 1 |
| Insecta | Hymenoptera | Nylanderia sp. | Introduced | 26 |
| Insecta | Hymenoptera | Nylanderia steinheili | Introduced | 5 |
| Insecta | Hymenoptera | Orasema costaricensis | Introduced | 8 |
| Insecta | Hymenoptera | Oxybelus schusteri | Introduced | 15 |
| Insecta | Hymenoptera | Parasierola sp. |  | 2 |
| Insecta | Hymenoptera | Paratrechina longicornis | Introduced | 845 |
| Insecta | Hymenoptera | Polistes versicolor | Introduced | 445 |
| Insecta | Hymenoptera | Scelionidae sp. 1 |  | 3 |
| Insecta | Hymenoptera | Scolebythidae sp. 1 |  | 2 |
| Insecta | Hymenoptera | Solenopsis geminata | Introduced | 36 |
| Insecta | Hymenoptera | Tapinoma melanocephalum | Introduced | 225 |
| Insecta | Hymenoptera | Tetramorium bicarinatum | Introduced | 82 |
| Insecta | Hymenoptera | Venturia canescens | Introduced | 1 |
| Insecta | Hymenoptera | Wasmannia auropunctata | Introduced | 421 |
| Insecta | Hymenoptera | Xylocopa darwini | Endemic | 875 |
| Insecta | Lepidoptera | Aetole galapagoensis | Endemic | 8 |
| Insecta | Lepidoptera | Agraulis vanillae | Endemic | 80 |
| Insecta | Lepidoptera | Amyna insularum | Endemic | 2 |
| Insecta | Lepidoptera | Anomis sp. |  | 20 |
| Insecta | Lepidoptera | Anticarsia gemmatalis | Native | 15 |
| Insecta | Lepidoptera | Asciodes gordialis | Introduced | 36 |
| Insecta | Lepidoptera | Atteva hysginiella | Endemic | 215 |
| Insecta | Lepidoptera | Cyclophora sp.1 |  | 7 |
| Insecta | Lepidoptera | Diaphania sp. |  | 2 |
| Insecta | Lepidoptera | Disclisioprocta stellata | Introduced | 90 |
| Insecta | Lepidoptera | Epidromia sp. |  | 23 |
| Insecta | Lepidoptera | Erinnyis ello encantada | Endemic | 170 |
| Insecta | Lepidoptera | Galagete sp.1 |  | 6 |
| Insecta | Lepidoptera | Heliodines galapagoensis | Endemic | 7 |
| Insecta | Lepidoptera | Hellinsia cristobalis | Endemic | 4 |
| Insecta | Lepidoptera | Hemiargus ramon | Introduced | 318 |
| Insecta | Lepidoptera | Hypena sp.1 |  | 6 |
| Insecta | Lepidoptera | Lantanophaga pusillidactyla | Introduced | 1 |
| Insecta | Lepidoptera | Lepidoptera sp. 1 |  | 9 |
| Insecta | Lepidoptera | Leptotes parrhasioides | Endemic | 273 |
| Insecta | Lepidoptera | Leucania cooperi | Endemic | 2 |
| Insecta | Lepidoptera | Loxomorpha cf. cambogialis | Native | 1 |
| Insecta | Lepidoptera | Manduca rustica | Endemic | 9 |
| Insecta | Lepidoptera | Melipotis harrisoni | Endemic | 3 |
| Insecta | Lepidoptera | Melipotis indomita | Native | 3 |
| Insecta | Lepidoptera | Neohelvibotis sp. 1 | Endemic | 8 |
| Insecta | Lepidoptera | Neohelvibotis sp. 2 |  | 1 |
| Insecta | Lepidoptera | Neohelvibotis sp. 3 |  | 3 |
| Insecta | Lepidoptera | Omiodes indicata | Introduced | 3 |
| Insecta | Lepidoptera | Ommatochila mundala | Native | 2 |
| Insecta | Lepidoptera | Oxydia lignata | Endemic | 1 |
| Insecta | Lepidoptera | Paectes arcigera | Introduced | 1 |
| Insecta | Lepidoptera | Perigonia lusca | Native | 1 |
| Insecta | Lepidoptera | Phoebis sennae | Native | 335 |
| Insecta | Lepidoptera | Psara chathamalis | Endemic | 4 |
| Insecta | Lepidoptera | Pseudoplusia includens | Native | 486 |
| Insecta | Lepidoptera | Pterophoridae |  | 4 |
| Insecta | Lepidoptera | Pterophoridae sp. 1 |  | 38 |
| Insecta | Lepidoptera | Pterophoridae sp. 2 |  | 9 |
| Insecta | Lepidoptera | Pyralidae sp. 1 |  | 6 |
| Insecta | Lepidoptera | Pyralidae sp.2 |  | 7 |
| Insecta | Lepidoptera | Pyrausta panopealis | Introduced | 57 |
| Insecta | Lepidoptera | Pyrausta sp. |  | 3 |
| Insecta | Lepidoptera | Spoladea recurvalis | Introduced | 145 |
| Insecta | Lepidoptera | Spragueia margana | Introduced | 9 |
| Insecta | Lepidoptera | Spragueia sp.1 |  | 2 |
| Insecta | Lepidoptera | Syngamia florella | Introduced | 155 |
| Insecta | Lepidoptera | Tineidae sp.1 |  | 1 |
| Insecta | Lepidoptera | Tota galdinella | Endemic | 2 |
| Insecta | Lepidoptera | Urbanus dorantes | Endemic | 696 |
| Insecta | Lepidoptera | Utetheisa ornatrix | Native | 4 |
| Insecta | Lepidoptera | Zale obsita | Native | 5 |
| Insecta | Odonata | Ischnura hastatum | Native | 1 |
| Insecta | Orthoptera | Acrididae |  | 2 |
| Insecta | Orthoptera | Acrididae sp.2 |  | 1 |
| Insecta | Orthoptera | Anaulocomera darwinii | Endemic | 10 |
| Insecta | Orthoptera | Gryllus sp. |  | 4 |
| Insecta | Orthoptera | Jarmilaxipha ecuadorica | Introduced | 4 |
| Insecta | Thysanoptera | Thysanoptera sp. 1 |  | 1 |
| Reptilia | Squamata | Microlophus bivittatus | Endemic | 1 |

**Figure S1.** Illustration of the entire pollination network, comprising data from the two islands (Santa Cruz and San Cristóbal), the three habitats (arid, transition and humid zones) and the two seasons (hot and cold). Data from 2010 and 2011 are also pooled. Plant species are depicted at the bottom of the network whereas pollinators are at the superior part of it. Alien (A) plants and their links are represented in red colour to illustrate the magnitude of the interactions in which they are involved, whereas endemic (Nze) and non-endemic natives (Nt) are represented in black and gray colour, respectively.

**Figure S2**. Mean (+ 1 S.E.) of network metrics showing differences between the two seasons across habitats for each study island in the hot season of 2011. Data on interaction evenness (IE) are shown for comparison with data from Figure 2 (2010 data), although differences across habitats were not significant this year. For each island, bars with the same letters indicate no differences across habitats (P > 0.05).


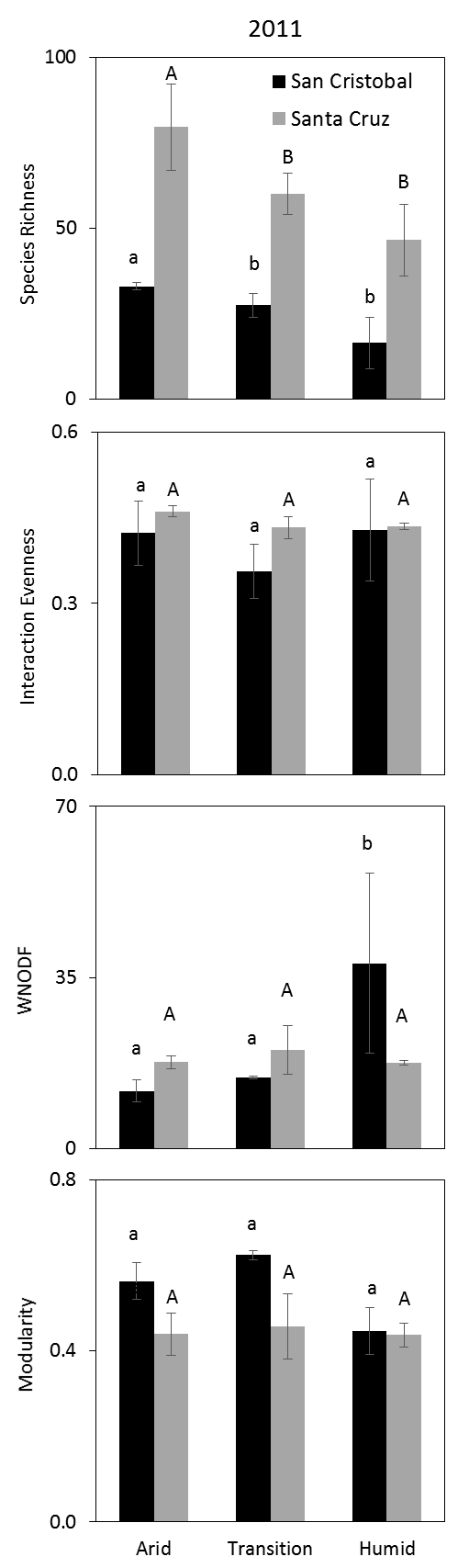


**Figure S3.** Mean (+ 1 S.D.) of the species-level parameters analysed in this study, for both pollinators and plants, showing differences among species from different habitats for the two islands, and the two seasons of 2010. Bars with the same letters on each graph indicate no differences across habitats (P > 0.05).


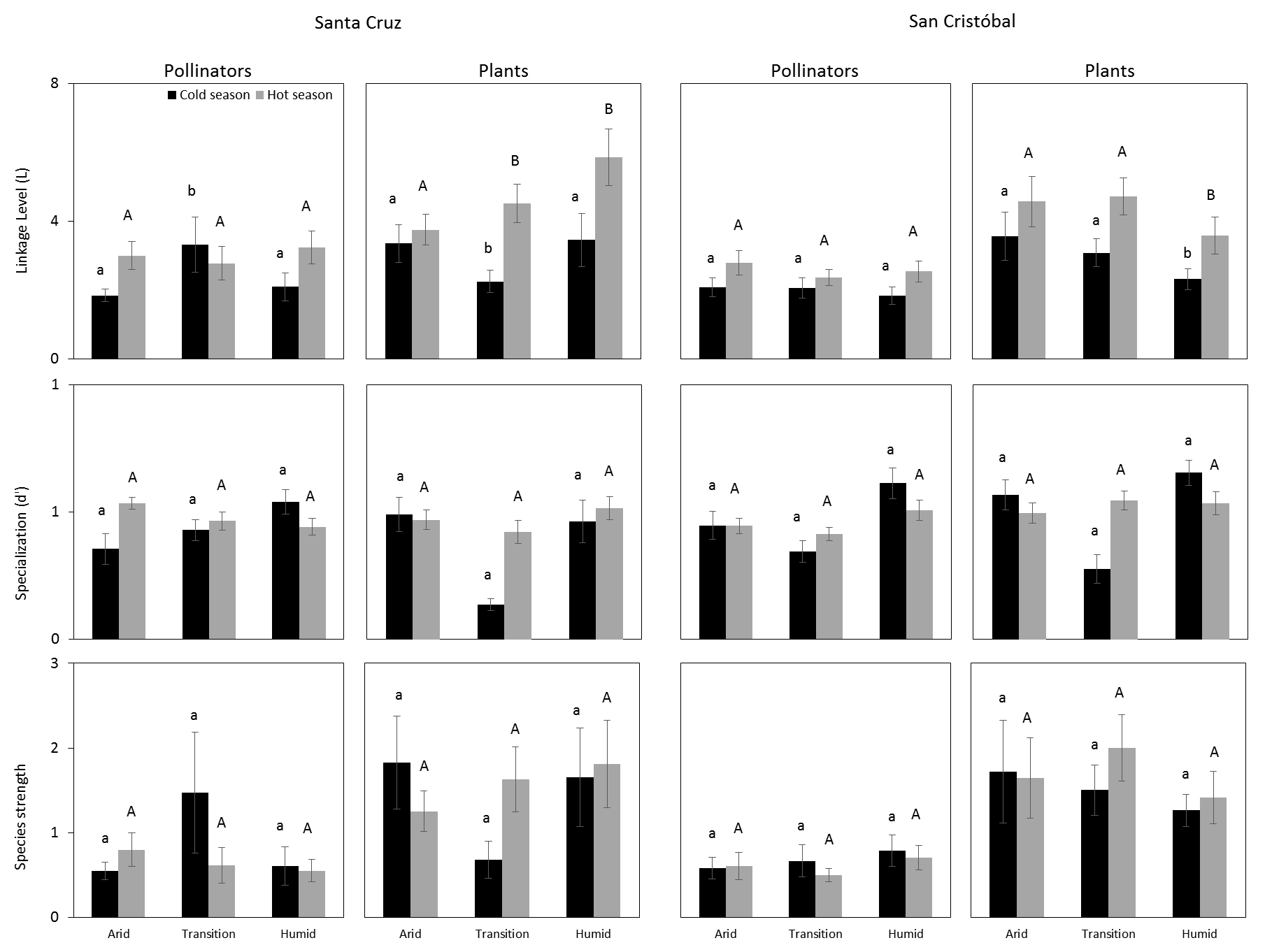

Supplement: Additional Information [file supp_plv068_plv068supp.docx]
